# A case report of steroid-refractory bullous pemphigoid induced by immune checkpoint inhibitor therapy

**DOI:** 10.3389/fimmu.2022.1068978

**Published:** 2023-01-04

**Authors:** Shasha Guan, Linlin Zhang, Junyan Zhang, Wenjing Song, Diansheng Zhong

**Affiliations:** ^1^ Department of Medical Oncology, Tianjin Medical University General Hospital, Tianjin, China; ^2^ Department of Dermatology, Tianjin Medical University General Hospital, Tianjin, China; ^3^ Department of Pathology, Tianjin Medical University, Tianjin, China

**Keywords:** immune checkpoint inhibitor, bullous pemphigoid, lung carcinoma, pembrolizumab, intravenous immunoglobulin

## Abstract

The widespread use of immune checkpoint inhibitors in several malignancies has revealed new immune-related adverse events. Bullous pemphigoid (BP) is an antibody-driven autoimmune disease characterized by skin inflammation and fluid-filled bullae. Herein, a 69-year-old man with lung squamous cell carcinoma developed multiple vesicles and tense bullae 3 weeks after the initiation of a programmed death-1 (PD-1) inhibitor, pembrolizumab, and chemotherapy. Biopsy revealed a subepidermal bulla with lymphocytic and eosinophil infiltration, and immunohistochemical studies predominantly showed CD4^+^ cells, a few CD8^+^ cells, and the occasional CD20^+^ lymphocyte. The serum anti-BP180 antibody level, as well as the interleukin-6 and interleukin-10 levels, were elevated compared to the lower levels of tumor necrosis factor-α. Eosinophil levels were high and consistent with the development of blisters. A diagnosis of BP associated with PD-1 inhibitor therapy was made, and the Common Terminology Criteria for Adverse Events classification was grade 3. Immunotherapy was permanently discontinued, and the patient’s bullous lesions failed to react to high-dose systemic corticosteroids combined with minocycline and niacinamide. Intermittent blister recurrence occurred in 2 months, eventually improving with the administration of two courses of intravenous immunoglobulin. At 5 weeks of follow-up, the patient’s tumor was reduced on a computed tomographic scan. Despite stable BP treatment, however, he repeatedly developed complications due to the complexity of his underlying disease and could not be treated with anti-tumor therapy. Early recognition and management of serious immune-related bullous dermatologic toxicity are essential for patient safety.

## 1 Introduction

Cancer immunotherapy, particularly immune checkpoint inhibitor (ICI) therapy, has revolutionized the treatment of malignancies. These monoclonal antibodies are directed against the inhibitory immune receptors cytotoxic T-lymphocyte-associated protein-4 (CTLA-4) and programmed death-1 (PD-1) and enhance the immune function of T-cells, thus mobilizing the body’s immune cells to selectively target cancer cells.

Given their unique mechanism of action, ICIs can also act on a variety of non-pathological human cell types, resulting in immune-related adverse events (irAEs) notably mediated by the triggering of cytotoxic CD4^+^/CD8^+^ T-cell activation. Distinct from the effects of traditional chemotherapy, irAEs tend to have a relatively delayed onset and are inflammatory or autoimmune in nature (1).

Dermatologic toxicities are the most prevalent irAEs associated with ICIs. Incidences of all grades of dermatologic toxicity range from 37%–70% for ipilimumab and 17%–40% for PD-1/programmed death-ligand 1 (PD-L1) inhibitors, respectively. Meanwhile, rates of high-grade dermatologic irAEs range from 1%–3% (2). More than 1/3 of treated patients present mainly with a maculopapular rash and pruritus. The antibody-driven autoimmune disease bullous pemphigoid (BP) induced by ICI therapy is a rare cutaneous irAE. BP is typically less severe than other cutaneous reactions such as toxic epidermal necrolysis (TEN) and stevens-johnson syndrome (SJS) (3). Whereas, for severe or life-threatening BP and all cases of SJS/TEN, hospitalization and permanent ICI therapy discontinuation are required. We present here a severe case of a patient with steroid-refractory BP that began shortly after initiating treatment with the PD-1 inhibitor pembrolizumab.

## 2 Case presentation

A 69-year-old man with lung squamous cell carcinoma began immunotherapy treatment with pembrolizumab (200 mg) and chemotherapy (albumin-bound paclitaxel) in February 2021 due to 60% PD-L1 expression and a poor Eastern Cooperative Oncology Group (ECOG) performance status of 2. He had received no prior treatment for the cancer. His medical history included diabetes, chronic obstructive pulmonary disease, and chronic eczema of the lower limbs. He was treated with oral hypoglycemic agents (metformin, repaglinide, and acarbose). No regular medication was given for the chronic eczema. He reported no history of autoimmune disorders. He had been a smoker for 1,000 pack-years without quitting.

**Figure 1:**
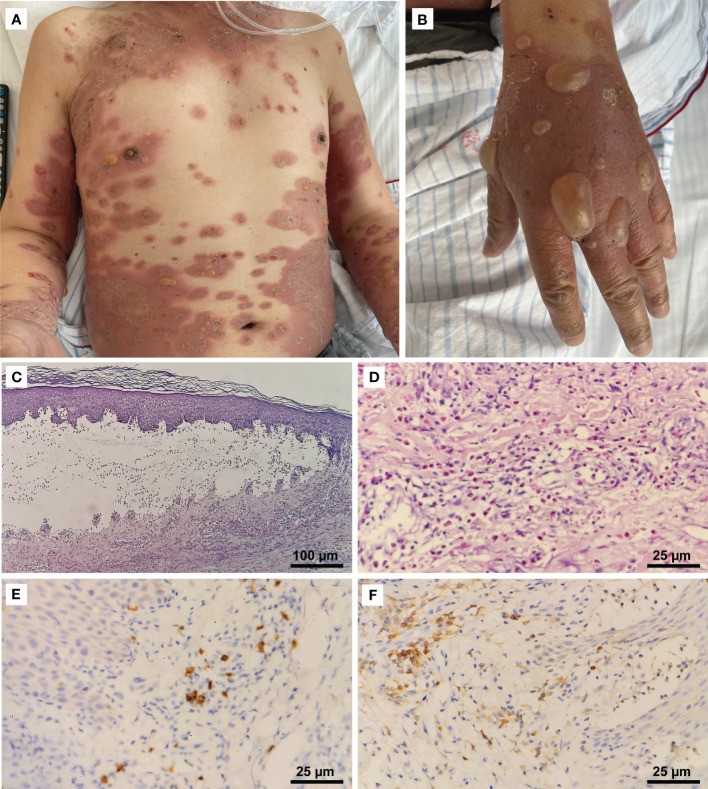
Clinical manifestations and histopathological features. **(A)** Erythematous patches and tense bullae on the patient’s trunk and extremities (CTCAE grade 3). **(B)** Numerous large tense bullae on the hand. **(C)** Histopathologic examination of a skin lesion on the right hand revealed a subepidermal blister cavity (H&E, 100×). **(D)** Eosinophils infiltrated the upper and mid-dermis (400×). Immunostaining revealed **(E)** a predominantly CD4^+^ (400×) lymphocytic infiltrate, with **(F)** a few CD8^+^ cells (400×) and the occasional CD20^+^ cell.

In the second week of treatment (9 days after the initiation of pembrolizumab and albumin-bound paclitaxel), the patient complained of intense itching initially, then developed extensive erythematous papules and plaques on his trunk and proximal part of the limbs. No improvement was seen with topical corticosteroids. Multiple vesicles and tense bullae, some with adjacent erosions and crusting, developed on his trunk and extremities following discontinuation (∼22 days) of pembrolizumab (**Figures 1A, B**). No oral or ocular mucosal involvement was noted. A biopsy examination of the bulla revealed a subepidermal blister cavity with associated dermal lymphocytic and eosinophil infiltration (**Figures 1C, D**). In addition, immunohistochemical studies of tissue sections predominantly showed CD4^+^ cells, a few CD8^+^ cells, and the occasional CD20^+^ lymphocytic infiltrate (**Figures 1E, F**). This revealed that CD4^+^-mediated immune responses in this patient were significantly enhanced. Laboratory examinations revealed that serum anti-BP180 antibodies (enzyme-linked immunosorbent assay [ELISA]) were elevated to >150 U/mL (normal range, <9 U/mL), while the anti-BP230 antibody (ELISA) concentration was below the detection limit (<5.0 U/mL). Eosinophil levels were high and consistent with the development of blisters. Interleukin-6 and interleukin-10 levels (flow cytometry) were elevated significantly to 37.76 pg/mL (reference range, 0–5.30 pg/mL) and 27.46 pg/mL (reference range, 0–4.91 pg/mL), respectively, compared to the lower level of tumor necrosis factor-α (0.63 pg/mL; reference range, 0–4.60 pg/mL).

As a consequence of these findings, drug-induced BP was diagnosed. Pembrolizumab was discontinued, and the patient’s bullae extended after 1 week of oral prednisone 40 mg daily (0.5 mg/kg) and topical high-potency topical steroids (halometasone). Given the patient’s severe bullous dermatitis (Common Terminology Criteria for Adverse Events [CTCAE] grade 3, i.e., affecting >30% of the body surface area, limiting self-care activities of daily living), immunotherapy was permanently discontinued, and he was treated with intravenous methylprednisolone 80 mg daily (1 mg/kg) augmented with minocycline and niacinamide.

The patient’s erythema became shallow; however, the coverage area was enlarged, and new active bullous lesions appeared. Thus, he underwent another skin biopsy of the edge of the erythema 5 weeks after his last dose of pembrolizumab. Pathology still confirmed BP, with CD4^+^ lymphocytic infiltrates predominating.

Due to his persistent symptoms, intravenous immunoglobulin (IVIG) was administered at 30 g (400 mg/m^2^) once daily for 5 days, resulting in rapid clinical improvement. Control of the BP was subsequently maintained with 40 mg of methylprednisolone daily.

**Figure 2:**
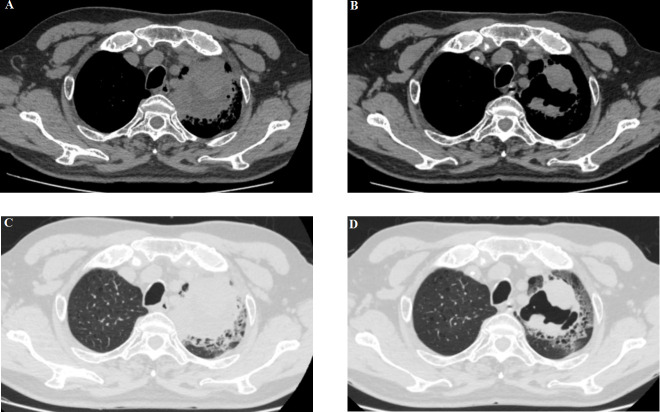
**(A, C)** A baseline computed tomography scan taken before treatment with ICI therapy, demonstrating the left lung mass. **(B, D)** A computed tomography scan taken at the time of BP development (6 weeks after pembrolizumab discontinuation), showing a partial response.

Meanwhile, the patient’s respiratory symptoms improved after immunotherapy. At 5 weeks of follow-up, the left lung mass showed partial response (PR) and became an irregularly cave on a computed tomographic scan (**Figure 2**). However, the patient repeatedly developed complications such as pneumonia, heart failure, blood glucose instability, and type II respiratory failure due to the complexity of his underlying disease and was treated with prolonged courses of antibiotics; thus, he could not be treated with anti-tumor therapy.

**Figure 3:**
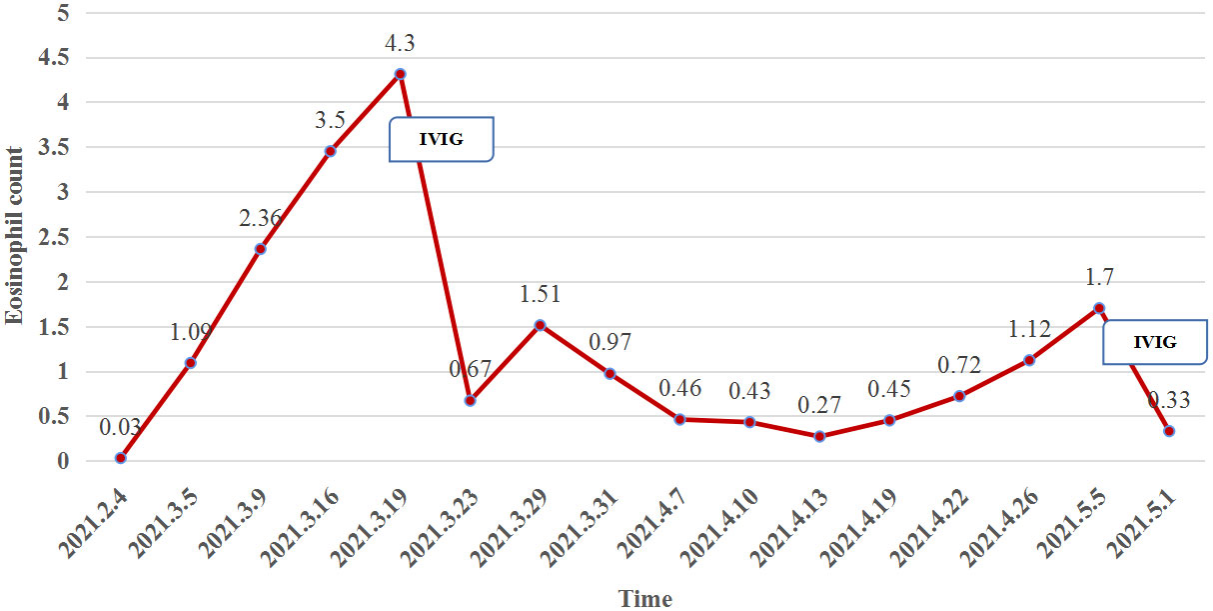
The eosinophil count (reference range, 0.02–0.05×10^9^/L) in the peripheral blood was concomitantly increased with the activity of the vesicles but plummeted after IVIG administration.

**Figure 4:**
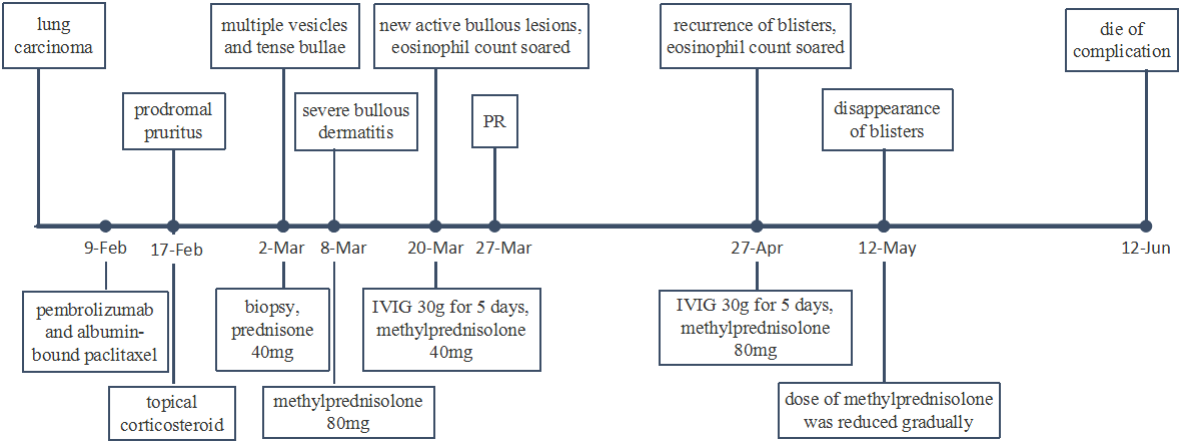
Case timeline. PR, partial response.

At week 11 (40 days after IVIG administration), the patient developed new vesicles on the anterior thorax and upper limbs again. Eosinophil count in the peripheral blood was concomitantly increased with the activity of the vesicles (**Figure 3**). He was treated with a second cycle of IVIG, and new blister formation ceased. Unfortunately, the patient died of gastrointestinal bleeding on June 12, 2021 (17 weeks after ICI discontinuation). The case timeline is shown in **Figure 4**.

A genetic analysis of the patient’s blood (via next-generation sequencing) suggested heterozygosity of HLA-A*02:07, HLA-A*11:01, HLA-B*13:01, HLA-B*46:01, HLA-C*01:02, and HLA-C*03:04.

## 3 Discussion

BP is an autoimmune blistering skin disease characterized by an autoimmune response to the hemidesmosomal protein BP180 within the dermal–epidermal junction that clinically manifests as tense blisters and erosions on the skin. Its typical histological appearance is subepidermal blisters with a dense dermal inflammatory infiltrate mainly consisting of eosinophils and neutrophils (4). Direct immunofluorescence of BP lesions shows deposits of immunoglobulin G and/or C3 along the basement membrane zone. The diagnosis of BP relies on lesion biopsy for hematoxylin and eosin (H&E) staining, perilesional biopsy for direct and indirect immunofluorescence microscopy, and anti-BP180/BP230 ELISA (5). Immune imbalance and antibody generation are the key pathophysiologies of autoimmune bullous diseases—that is, a breakdown of T- and B-cell tolerance to BP antigens leads to auto-antibody production and subsequent blister induction. Recently, many studies have found that T-cell subsets, which are critical players in autoimmunity, exhibit a range of abnormalities and drive immunopathogenesis and skin inflammation in BP (6). Genetic factors also seem to play an important role in a patient’s predisposition to BP. Many polymorphisms of HLA alleles, especially HLA-DQB1*03:01, have been identified in patients with BP across several populations. This allele may thus be involved in the presentation of immunodominant epitopes of BP180 to autoreactive T-cells in BP (7). This association has not been explicitly studied in patients with immunotherapy-induced BP; however, if such an association could be established, extended monitoring for BP may be necessary for some patients being treated with PD-1/PD-L1 inhibitors.

PD-1 or PD-L1 inhibitor use is a known risk factor for drug-induced BP. The latency of bullous disorders due to immunotherapy is generally longer than that of other cutaneous toxicities. In most cases, BP onset is noted concurrently with medication use within 6–8 months of drug initiation (8–10). In this case, however, the bullae first appeared earlier, approximately 3 weeks after the patient’s last infusion. Pruritus and other non-specific cutaneous findings may be the only precursors of immunotherapy-induced BP, as was observed here. Therefore, a biopsy is generally required, including both lesional and perilesional sampling of the patient’s skin. Given the pharmacodynamics and pharmacokinetics of ICIs, the responses may develop after many weeks or months of immunotherapy, with benefits lasting even after discontinuation. Thus, ICI-induced BP is usually not self-limiting.

ICIs bind to immune checkpoint receptors to reverse the inhibition of T-cell activation, thus restoring anti-tumor immune responses. Removal of this inhibition may also result in both generalized and tissue-specific inflammation, collectively known as irAEs. IrAEs are thought to principally arise from the PD-1/PD-L1 pathway’s protection against T-cell–mediated autoimmunity (11). However, B-cells that secrete antibodies may also play a role in the development of irAEs (12). The causal relation between BP and ICIs remains unclear. After ICI usage, epithelial cells and nearby cells may be attacked by the immune system, resulting in the generation of high levels of CD4 helper T-cell cytokines or increased migration of cytolytic CD8^+^ T-cells within normal tissues. Previously inhibited T-cells could amplify and improve the activity of the immune system, resulting in the release of auto-antibodies from previously depressed B-cells, thereby triggering irAEs. This reaction may raise a pre-existing auto-antibody response or represent an entirely new process caused by immunotherapy treatment (13). BP180 is an antigen expressed on the surface of malignant melanocytic tumor cells, non–small-cell lung cancer cells, and the basement membrane of the skin. Auto-antibody production against BP180 can weaken the dermal–epidermal junction, leading to a subepidermal cleft of the basement membrane zone.

In our case, though chemotherapy-related cutaneous toxicity cannot be excluded and paraneoplastic BP should be considered, BP induced by anti–PD-1 therapy was favored given the persistent recurrence of bullae over a 2-month period, T lymphocytic infiltrates, and stability of the patient’s malignancy with treatment. Immunostaining revealed mostly CD4^+^ T-cells, with a few CD8^+^ cells and the occasional CD20^+^ cell, indicating that a T-cell–mediated immune response predominated in this context. This further confirms the presence of immune-related adverse reactions. Perhaps the refractoriness of our patient’s irAE was attributable due to the combined presence of the B-cell–mediated immune response, which may reflect the consequences of unsuppressed cell-mediated immunity, even through the innate immune component of helper T-cells.

Eosinophilic lesional infiltrates and highly peripheral eosinophilia levels are well-known features of BP, and anti-BP180 immunoglobulin E results in essential eosinophil degranulation and consequent blister formation (14). In our case, the change in eosinophil count was consistent with blister development (**Figure 3**).

Drug-induced BP is characterized by spontaneous exacerbations, typically requiring treatment for 6–12 months involving mainly topical or systemic corticosteroids. Patients with severe bullous dermatitis (G3/4) should permanently discontinue immunotherapy and receive daily treatment with 1–2 mg/kg of prednisone/methylprednisolone. The addition of 1–2 systemic treatment options is often necessary, such as dapsone, azathioprine, mycophenolate mofetil, or doxycyclin. If no improvement is seen after 3 days, suggesting treatment resistance, rituximab and the anti–immunoglobulin E monoclonal antibody omalizumab may be considered (15). IVIG has been used to suppress a wide array of autoimmune and chronic inflammatory conditions. The immunomodulatory mechanisms of IVIG include modulating the activity and effector functions of B and T lymphocytes, impacting antigen presentation, pathogenic auto-antibodies, the complement system, and cytokines (16).

In most reported cases, BP during ICI therapy has been relatively mild. Systemic and topical corticosteroids were the most common treatments, whereas biologic and targeted agents were used predominantly in cases refractory to treatment with corticosteroids (10). Our patient exhibited severe and extensive bullous lesions refractory to high-dose corticosteroids (1 mg/kg). Despite combining therapy with minocycline and niacinamide, the patient’s BP still did not respond well. To clarify the immune microenvironment of BP, we performed immunostaining, which confirmed that the infiltrating lymphocytes were mainly CD4^+^ T-cells, with the occasional CD20^+^ T-cell also identified, further guiding the treatment of BP. IVIG was administered instead of the anti-CD20 monoclonal antibody rituximab. With the application of two cycles of IVIG, the bullae were finally controlled, and the hormone dose (40 mg of methylprednisolone daily) was gradually tapered off. Considering the tumor response, when BP was present, the patient’s tumor mass shrank significantly. A retrospective study suggested that patients who develop ICI-induced BP may experience improved tumor responses and survival outcomes compared to those who do not (17). In addition, retrospective data generally suggest that immunosuppressive therapy (primarily systemic corticosteroids) initiated after irAE onset does not appear to decrease ICI efficacy (18).

What clinicians are most concerned about is whether ICI therapy can be continued. The mean elimination half-life of both pembrolizumab and nivolumab is approximately 26 days, but the effects of PD-1 inhibitors almost certainly last longer. The persistence of lesions several months after ICI therapy discontinuation has also been reported (17). Significant irAEs often necessitate significant caution regarding the rechallenge of immunotherapy.

There are a few limitations to our study. Given its rarity, no disease-specific guidelines exist for the treatment of ICI-induced BP, and it remains uncertain whether immunohistochemical staining of lesion pathology could guide biological and targeted treatment of such. The current knowledge base of all aspects of ICI-induced BP is limited. Clinicians should be aware of the early recognition and grading of irAEs, timely treatment, and individualized management with immunosuppression and ICI administration. Even following ICI therapy discontinuation, aggressive management to prevent extensive disease with high morbidity is pivotal to optimize clinical efficacy and safety.

## Data availability statement

The original contributions presented in the study are included in the article/supplementary material. Further inquiries can be directed to the corresponding author.

## Ethics statement

The studies were reviewed and approved by The Ethical Committee of Tianjin Medical University General Hospital (Ethical NO. IRB2022-WZ-208).The patients/participants provided their written informed consent to participate in this study. Written informed consent was obtained from the individual(s) for the publication of any potentially identifiable images or data included in this article.

## Author contributions

SG, LZ and DZ designed and wrote the initial draft of the manuscript. Images of histology slides were created and captioned by WS. SG, LZ, JZ and DZ revised the paper. DZ leads a multidisciplinary team to guide patient management of irAEs for oncology patients being treated with immune checkpoint inhibitors at Tianjin Medical University General Hospital. All authors contributed to the article and approved the submitted version.
